# Cognitive function of older adults engaging in physical activity

**DOI:** 10.1186/s12877-020-01620-w

**Published:** 2020-07-02

**Authors:** Monisha Ingold, Nikki Tulliani, Chetwyn C. H. Chan, Karen P. Y. Liu

**Affiliations:** 1 School of Health Sciences, Western Sydney University, Locked Bag 1797, Penrith, NSW 2751 Australia; 2 Translation Health Research Institute, Western Sydney University, Locked Bag 1797, Penrith, NSW 2751 Australia; 3 Department of Rehabilitation Sciences, The Hong Kong Polytechnic University, Hung Hom, Hong Kong; 4 University Research Facility in Behavioural and Systems Neuroscience, The Hong Kong Polytechnic University, Hung Hom, Hong Kong

**Keywords:** Older adults, Physical activity, Cognitive function, Self-regulation

## Abstract

**Background:**

Physical activity can be classified as open-skilled or closed-skilled. Open-skilled physical activity, such as tennis, require participants to perform within a dynamic setting and respond to unpredictable and frequent environmental changes throughout the activity. Closed-skilled types of physical activity, such as swimming, are predictable and self-directed. However, the benefits of cognitive function in these two types of physical activities to older adults are unknown. This study examined the effects of participation in open- and closed-skilled physical activity on the cognitive function of older adults.

**Methods:**

The study recruited a total of 61 participants aged 65 years and over. Participant recruitment was achieved by distributing flyers asking for volunteers in various sports venues. Participants self-reported to be without medical conditions affecting their physical and cognitive function. All participants underwent a two-hour assessment session involving the completion of seven standardised cognitive function assessments, which were used to assess a range of cognitive function.

**Results:**

Overall mean scores across all of the assessments showed superior performance for the open- or closed-skilled participants when compared with the no-physical-activity group. The results of 61 adults who participated in this study showed that closed-skilled physical activity was associated with better selective attention and visuospatial function while open-skilled physical activity was associated with better inhibition and cognitive flexibility function. No significant difference in self-regulation ability was found between the open- or closed-skilled groups.

**Conclusions:**

Open-skilled physical activity was associated with better inhibition, visual tracking, and cognitive flexibility while closed-skilled physical activity was associated with better selective attention and visuospatial perception. The findings have important practical implications for the health and quality of life of ageing populations, knowing which particular types of physical activity might affect the cognitive function.

## Background

As the ageing population is expected to almost triple by 2050 [1], there is a growing need for strategies to militate against common health issues associated with ageing to reduce the burden on the health system [2]. While the ageing process is understood to alter people’s cognitive function, with variability existing across both age and sex [3], regular participation in physical activity has been correlated with positive changes to brain structure and volume, such as an increase in white matter and parietal lobe gray matter volume [4] and hippocampal and basal ganglia volume [5, 6], and with improvement in a wide range of cognitive functioning skills in older adults [3, 7–12]. Physical activity is understood to promote cellular processes that influence the brain’s neuroplasticity, particularly those processes involving the synthesis of brain-derived neurotrophic factor (BDNF) in the hippocampus [13]. BDNF production provides a range of important functions for learning including neuronal and synaptic growth and differentiation, neuronal protection, and synaptic transmission [13, 14]. As a result, participating in physical activity has been shown to provide protection against age-related cognitive function loss [e.g. [4–6, 15, 16].

One area of cognitive function that is known to decline as a consequence of the ageing process is executive function – the cognitive processes, primarily linked to the prefrontal cortex of the brain, which guide and regulate purposeful, goal-directed behaviour [17, 18]. These cognitive processes include planning, interference control, dual-task coordination [17], shifting, updating, inhibition [18] and working memory [19], all of which play important roles in the performance of everyday functions from basic activities of daily living, such as grooming, through to more complex instrumental activities such as managing personal finances [20]. Each cognitive function serves a specific purpose but is used in combination with others to perform daily functions [21]. For example, effective dual-task coordination relies on both effective inhibitory function, which is the ability to suppress an irrelevant stimulus or inappropriate reaction, and working memory function, which is the temporary storing of information to be retrieved for learning and completing tasks [22]. Due to the significance of cognitive function processes in performing daily tasks, cognitive dysfunction can be associated with a decline in everyday functioning, which is particularly associated with age-related neurodegenerative diseases such as Alzheimer’s disease [23].

One important goal-directed behaviour in the cognitive function is self-regulation. Self-regulation refers to the processes involved in managing thoughts, feelings, and behaviours [24]. These processes are essential for people’s ability to demonstrate effective human behaviour [25] and include the capacity to plan, think flexibly, initiate appropriate behaviour, and inhibit inappropriate behaviour [26]. Self-regulation serves two key functions for effective daily functioning. Firstly, it helps people overcome challenges and achieve desired outcomes, such as the pursuit of long-term goals at the expense of short-term gratification [27]. Secondly, it is used for adapting goals in order to avoid consequences that may be psychologically distressing and protect an individual’s sense of self by, for example, ignoring the impulse to carry out decisions that pose significant risk [28]. As unsuccessful implementation of self-regulatory behaviour poses a risk to effective daily functioning [27], it is an important area of focus as it affects older adults’ quality of life and overall health [28].

Physical activity can be classified as open-skilled or closed-skilled [29]. Open-skilled physical activity, such as tennis, basketball, and fencing, requires participants to perform within a dynamic setting and respond to unpredictable and frequent environmental changes throughout the activity [30, 31]. In contrast, closed-skilled types of physical activity, such as swimming, running, or yoga, are not dependent upon rapid changes in the environment and are instead predictable and self-directed [31]. Both types of physical activity require self-regulation. However, while participation in open-skilled physical activity demands more cognitive effort from individuals because they must provide immediate responses to unpredictable environmental cues [17], closed-skilled physical activity demands attention on particular, often repetitive body movements for effective performance [32]. With the various forms and the need to interact with the environment in both open- and closed-skills physical activity, there are reports highlighting the association with memory [33] and especially object location memory [34]. While any specific benefits that closed-skilled as opposed to open-skilled physical activity may provide have not been identified, the vast majority of research has found there to be promising effects for a range of cognitive function from engagement in open-skilled physical activity [for example, [29, 35, 36]. However, the literature is primarily limited to younger populations, particularly children [37–39] and young adults [31, 40–42], with one frequently cited study regarding middle-aged adults [43]. Although three studies have compared open- and closed-skilled physical activity in older populations, only the executive function subcomponents of inhibitory function [17], task-switching [30] and visuospatial working memory [44] have been investigated. Within the limited scope of these studies, it was found that due to open-skilled physical activity demanding more immediate responses than closed-skilled physical activity, open-skilled participants exhibited superior performance in the cognitive function under examination [17, 30, 44].

Additionally, while the association between physical activity and self-regulation function in older adults has been studied, the focus has been on the extent to which self-regulation acts as a determinant of older adults’ maintenance of health and physical activity behaviours rather than on the effects of physical activity engagement on self-regulation functioning ability [28, 45–50]. There is, therefore, a major role in our understanding of whether physical activity participation plays a role in supporting self-regulation functioning in older adults, potentially enabling greater daily functioning and quality of life, and whether the type of physical activity also has any significant role in this.

### The present study

This study intends to address these identified gaps in the literature by investigating the effects of open- versus closed-skilled physical activity on self-regulation and a wider variety of cognitive functions specifically among older people. The above-mentioned research underpins three hypotheses that were formulated for this study. Firstly, we expected to find better cognitive function in participants who engaged in either open- or closed-skilled physical activity when compared with the no-physical activity participants. Secondly, we predicted there would be a greater effect on the cognitive function for the open-skilled group compared with both the closed-skilled and non-physical activity groups due to the apparent additional benefits from participating in these more cognitively-demanding types of physical activity [17, 30, 31, 42–44]. Finally, it was hypothesised that more frequent engagement in physical activity would positively impact the cognitive function of the open- and closed-skilled groups.

## Methods

A quantitative, cross-sectional research design was implemented for this study.

### Participants

A total of 61 community-dwelling older adults aged 64 to 87 (mean age = 71.93, SD = 5.50) were recruited by convenience sampling method over a six-month period. Recruitment was conducted through flyers displayed in various sports venues in the greater Sydney region.

All reported being healthy without a medical history affecting their physical and cognitive function. Twenty-one engaged in open-skilled sports for at least 1 h per week for the previous year, 15 in closed-skilled sports, and 25 with no engagement in physical activity (Table 1). No significant difference in age (*P* = .71), sex (*P* = .59) and education level (*P* = .90) was found among the three groups.

**Table 1:** Physical Activity Types (*N* = 61)

	Type	Frequency	Percentage
Open-Skilled (*n* = 21)	Golf	9	14.8
	Lawn Bowls	9	14.8
	Tennis	2	3.3
	Basketball	1	1.6
Closed-Skilled (*n* = 15)	Tai Chi	4	6.6
	Gym	1	1.6
	Line Dancing	2	3.3
	Walking	4	6.6
	Swimming	3	4.9
	Cycling	1	1.6
No Physical Activity (*n* = 26)		26	42.6
	Total	61	100

### Procedure

Self-reported information was taken from all participants regarding demographic details, physical activity participation consisted of the type of activity and the number of years of engagement, as well as the total number of hours and sessions per week for each physical activity. Participants then underwent a 1–2 h session involving the completion of five standardised cognitive assessments and two self-regulation questionnaires. The study obtained ethical approval from the Western Sydney University Human Research Ethics Committee (approval number H11724).

### Measures

The following standardised assessments were used to measure cognitive function.

#### Repeatable battery for the assessment of neuropsychological status

The immediate memory and delayed memory sections of the Repeatable Battery for the Assessment of Neuropsychological Status (RBANS) were administered in this study. This instrument has been used widely to detect and track the performance of a range of cognitive abilities in older adults [51].

#### Trail making test - parts a and B

Both parts of the Trail Making Test measure processing speed, visual scanning, and mental flexibility through timing participants’ ability to connect numbers from 1 to 25 (Trail Making A), and alternate between numbers and letters (Trail Making B) in ascending order [52]. Trail Making B is considered to have additional cognitive demands compared with Trail Making A [11, 53]. Participants achieve a better performance score in both parts based on the shortest time it takes to complete the tasks. Therefore, a smaller number in their assessment score indicates better performance in the assessment.

#### Benton Judgement of line orientation test

The Benton Judgement of Line Orientation Test measures visuospatial perception. Participants are required to judge the distance between a pair of lines in relation to a diagram of 11 lines displayed in a semi-circle formation. The test has been used among a wide range of older adults [54] and is considered to have strong overall psychometric properties [55].

#### Digit span forward and digit span backward

The Digit Span Forward measures attention and processing speed while the Digit Span Backward measures working memory by, in both sections, asking participants to recall a sequence of digits in the correct order [56]. Sequences begin with two digits and extend by one number after each successful attempt by the participant. The number of digits successfully recalled by the participants was reported. The test is used extensively among a diverse range of populations, including older adults [57].

#### Stroop color and word test

The Stroop Color and Word Test is divided into three parts: Stroop (Word) [Stroop (W)], Stroop (Color) [Stroop (C)] and Stroop (Color and Word) [Stroop (CW)] and measures information processing speed, selective attention and inhibition by requiring participants to read words and name colours as rapidly and correctly as possible within 45 s [58]. Within the task, participants must ignore the written colour word and state the colour of the ink, which requires them to simultaneously ignore some information while selectively attending to target information. As a result, it is considered to be an effective measure of attention and has been used extensively in older populations [8]. Only the T-scores of the assessment were analysed and reported in this study.

#### Self-regulation questionnaire

The Self-Regulation Questionnaire (SRQ) is a self-reporting tool containing 63 items on a 5-point Likert scale, which measures seven areas of self-regulation: receiving relevant information, evaluating the information, triggering change, searching for options, formulating a plan, implementing a plan and assessing a plan’s effectiveness. Instead of being domain-specific, this instrument measures general self-regulation ability and has been used extensively with adult populations [59].

#### Self-regulation of learning self-report scale

The Self-Regulation of Learning Self-Report Scale (SRL-SRS) is a self-reporting assessment tool containing 50 items. It measures the areas of planning, self-monitoring, evaluation, reflection, effort, and self-efficacy within multiple learning domains [60].

### Data analysis

Descriptive statistics on the demographic data and the results of the seven standardised assessments were analysed. Pearson correlations were used to assess the relationship between the cognitive function measures in the open-skilled, closed-skilled, and no physical activity participation groups, and between assessment results and participations in physical activity. The Multivariate Analysis of Variance (MANOVA) was used to explore the differences in cognitive function between the three participant groups. Multivariate Analysis of Co-Variance (MANCOVA) was used with age and sex as covariates in the analysis because the variance in cognitive function measures has been detected among different older age-groups [61] and between men and women [3]. Further analysis in the cognitive function measures between the participants in the open- and closed-skilled physical activity groups was completed using MANCOVA with age, sex, and participation in physical activity as covariates.

## Results

Table 1 shows the distribution of physical activity types from the open- and closed-skilled groups. The most common types of physical activity among the open-skilled group were golf (*n* = 9) and lawn bowls (*n* = 9) while Tai Chi (*n* = 4), walking (*n* = 4) and swimming (*n* = 3) were the most common types of physical activity among the closed-skilled group.

The demographics and mean scores of the three participant groups’ physical activity participation and standardised assessments are presented in Table 2. The mean age of the open-skilled group (*M* = 71.52, *SD* = 6.23), closed-skilled group (*M* = 71.33, *SD* = 5.19) and non-physical activity group (*M* = 72.64, *SD* = 5.16) were similar (*P* = .71). The open-skilled group (*M* = 17.16, *SD* = 16.94) had spent more years engaging in physical activity than the closed-skilled group (*M* = 12.27, *SD* = 12.15) but no significant difference was found (*P* = .22). The open-skilled group engaged in significantly more hours of physical activity per week (*M* = 3.33, *SD* = 1.23) than the closed-skilled group (*M* = 1.00, *SD* = .46) (*P* = .001). However, the closed-skilled group (*M* = 3.36, *SD* = 1.98) engaged in significantly more sessions per week than the open-skilled group (*M* = 1.76, *SD* = .89) (*P* < .001).

**Table 2:** Demographics Information and Assessment Results

	Open-skilled (*n* = 21; Female = 9; Male = 12)		Closed-skilled (*n* = 15; Female = 9; Male = 6)		No physical activity (*n* = 25; Female = 12; Male = 13)		Total Sample (*n* = 61; Female = 30; Male = 31)	
	Mean (SD)	Range	Mean (SD)	Range	Mean (SD)	Range	Mean (SD)	Range
Age	71.52 (6.23)	65–87	71.33 (5.19)	64–83	72.64 (5.16)	65–84	71.93 (5.50)	64–87
Physical activity participation (years)	17.16 (16.94)	1–53	12.27 (12.15)	0–37			8.93 (13.78)	0–53
Duration of Physical Activity Session (hours/week)	3.33 (1.23)	1–5	1.00 (.46)	0–2			1.39 (1.65)	0–5
Frequency of Physical Activity (sessions/week)	1.76 (.89)	1–4	3.36 (1.98)	0–7			1.40 (1.71)	0–7
RBANS IM Index Score	92.43 (17.45)	65.00–126.00	**96.47** (13.23)	78.00–120.00	91.00 (16.12)	53.00–114.00	92.84 (15.85)	53.00–126.00
RBANS DM Index Score	**95.33** (12.93)	60.00–110.00	92.73 (14.25)	64.00–112.00	95.16 (12.51)	60.00–116.00	94.62 (12.92)	60.00–116.00
Trail Making A	**31.69^** (10.51)	19.10–67.48	33.58 (12.26)	16.80–65.10	35.60 (11.26)	20.68–63.07	33.76 (11.20)	16.80–67.48
Trail Making B	**72.09^** (25.43)	33.30–142.22	72.47 (24.97)	31.15–123.00	86.58 (28.28)	40.34–169.26	78.12 (27.04)	31.15–169.26
Benton Judgement of Line Orientation	20.05 (7.12)	13.00–30.00	**22.60** (6.80)	13.00–30.00	15.12 (3.56)	11.00–26.00	18.65 (6.52)	11.00–30.00
Digit Span Forward	5.81 (1.86)	3.00–10.00	**6.33** (1.54)	3.00–8.00	5.28 (1.31)	3.00–7.00	5.72 (1.60)	3.00–10.00
Digit Span Backward	**4.38** (1.28)	2.00–7.00	4.20 (1.01)	3.00–6.00	3.92 (1.22)	2.00–7.00	4.15 (1.19)	2.00–7.00
Stroop (W) T-Scores	**50.33** (8.14)	35.00–66.00	44.20 (9.46)	28.00–62.00	42.76 (14.78)	4.00–69.00	45.72 (11.92)	4.00–69.00
Stroop (C) T-Scores	39.05 (13.84)	−2.00 – 59.00	**43.93** (10.60)	20.00–62.00	31.00 (19.43)	−16.00 – 53.00	36.95 (16.42)	−16.00 – 62.00
Stroop (CW) T-Scores	**52.95** (8.83)	40.00–71.00	47.80 (9.49)	30.00–71.00	45.68 (7.64)	34.00–62.00	48.70 (8.98)	30.00–71.00
SRL-SRS-Planning	**29.57** (4.64)	18.00–37.00	28.33 (6.41)	10.00–35.00	27.88 (7.12)	14.00–46.00	28.57 (6.13)	10.00–46.00
SRL-SRS-Self-Monitoring	**26.81** (4.35)	14.00–32.00	25.60 (4.08)	15.00–32.00	24.60 (5.40)	12.00–36.00	25.61 (4.77)	12.00–36.00
SRL-SRS-Evaluation	**33.48** (4.07)	27.00–40.00	31.33 (6.14)	22.00–40.00	30.08 (6.20)	14.00–40.00	31.56 (5.65)	14.00–40.00
SRL-SRS-Reflection	17.38 (6.87)	0–24.00	**17.67** (6.36)	7.00–25.00	15.20 (5.29)	5.00–23.00	16.56 (6.14)	0–25.00
SRL-SRS-Effort	32.14 (9.20)	0–40.00	**32.67** (4.27)	25.00–40.00	29.64 (6.62)	13.00–40.00	31.24 (7.20)	0–40.00
SRL-SRS-Self-Efficacy	30.62 (8.29)	0–39.00	**31.73** (4.33)	26.00–40.00	28.24 (6.35)	8.00–37.00	29.92 (6.75)	0–40.00
SRQ	**228.62** (19.99)	182.00–280.00	213.80 (60.61)	0–260.00	216.04 (22.65)	157.00–258.00	219.82 (35.18)	0–280.00

Highest mean scores in bold. *95%* Confidence Interval for Mean, *SD* standard deviation, ^ lower score indicates better cognitive score

*RBANS IM* Repeatable Battery for the Assessment of Neuropsychological Status: Immediate Memory, *RBANS DM* Repeatable Battery for the Assessment of Neuropsychological Status: Delayed Memory, *(W)* Word, *(C)* Colour, *(CW)* Colour-Word, *SRL-SRS* Self-Regulation of Learning Self-Report Scale, *SRQ* Self-Regulation Questionnaire

By reviewing the raw scores of the cognitive function between the open-skilled and closed-skilled groups (Table 2), the open-skilled group achieved better results in the RBANS Delayed Memory, Trail Making A, Trail Making B, Digit Span Backward, Stroop (W), Stroop (CW), the SRL-SRS components of Planning, Self-Monitoring, and Evaluation and the Self-Regulation Questionnaire (SRQ). However, the closed-skilled group performed more effectively in the RBANS Immediate Memory Index Score, the Benton Judgement of Line Orientation, Digit Span Forward, Stroop (C), and the SRL-SRS components of Reflection, Effort, and Self-Efficacy. The ‘no physical activity’ group did not achieve any mean scores that were better than either the open- or closed-skilled groups for any of the assessments.

When reviewing participants’ physical activity engagement (hours per week, sessions per week and years of participation), significant correlations (*P* < .05) were found between hours of physical activity engagement per week and cognitive function measures for the Benton Judgement of Line Orientation (visuospatial perception) (*r* = .28, *P* = .03), Digit Span Backward (working memory) (*r* = .31, *P* = .02), Stroop (W) (information processing and attention) (*r* = .29, *P* = .03) and Stroop (CW) (selective attention and inhibition) (*r* = .38, *P* = .003))(Table 3). The number of years participants engaged in physical activity was significantly correlated with results from the Benton Judgement of Line Orientation (*r* = .44, *P* < .001) and the Stroop (W) (*r* = .32, *P* = .01). The frequency of physical activity participation per week was significantly correlated with scores from the Benton Judgement of Line Orientation (*r* = .40, *P* = .001), the Digit Span Forward (attention and processing speed) (*r* = .33, *P* = .01) and Stroop (C) (*r* = .28, *P* = .03).

**Table 3:** Pearson Correlations between Participation in Physical Activity and Cognitive Functions (*N* = 61)

	1	2	3	4	5	6	7	8	9	10	11	12	13	14	15	16	17	18	19	20
1. Hours per week	–																			
2. Sessions per week	.60**	–																		
3. Number of years	.54**	.45**	–																	
4. RBANS IM Index	−.02	.12	−.01	–																
5. RBANS DM Index	.08	.16	−.11	.58**	–	–														
6. Trail Making A	−.02	−.09	.00	−.17	−.10	–														
7. Trail Making B	−.12	−.23	.08	−.42**	−.36**	.53**	–													
8. Benton Judgement of Line Orientation	.28*	.40**	.44**	.03	−.06	.00	.09	**–**												
9. Digit Span Forward	.20	.33*	.23	.27*	.28*	−.19	−.18	.48**	–											
10. Digit Span Backward	.31*	.20	.11	.26*	.23	.02	−.18	.06	.36**	–										
11. Stroop (W)	.29*	.14	.32*	.20	−.00	−.21	−.20	.08	.31*	.33**	**–**									
12. Stroop (C)	.25	.28*	.14	.34**	.31*	−.26*	−.47**	−.09	.44**	.14	.39**	–								
13. Stroop (CW)	.38**	.19	.15	.25	.24	−.17	−.44**	−.03	.35**	.40**	.47**	.53**	–							
14. SRL-SRS-Planning	.06	.14	−.04	.29*	.35**	−.27*	−.29*	−.12	.13	.03	−.26*	.25	.10	–						
15. SRL-SRS- Self-Monitoring	.04	.06	.19	.14	.24	−.02	.02	.06	.13	−.06	−.02	.26*	.19	.56**	–					
16. SRL-SRS- Evaluation	.16	.03	.12	.21	.18	−.09	−.02	.02	.09	−.01	−.03	.23	.09	.43**	.67**	–				
17. SRL-SRS-Reflection	.18	.13	.07	−.06	−.18	.09	.08	.10	−.03	−.02	−.03	−.10	−.03	−.05	.10	.36**	–			
18. SRL-SRS-Effort	.13	−.05	−.01	.04	.11	.18	−.03	.01	−.15	−.21	−.02	.19	.11	.21	.40**	.44**	.29*	–		
19. SRL-SRS-Self-Efficacy	.18	.08	−.03	.16	.14	.10	−.06	−.08	−.32*	−.11	.01	.04	−.00	.14	.24	.27*	.28*	.66**	–	
20. SRQ	.09	−.25	−.08	−.01	−.10	−.17	−.07	−.11	−.11	.06	.05	.09	−.02	.12	.02	.09	−.14	.12	.07	–

Coefficients printed in bold are significant: ** Correlation is significant at the 0.001 level (2-tailed); * Correlation is significant at the 0.05 level (2-tailed)

*RBANS IM* Repeatable Battery for the Assessment of Neuropsychological Status: Immediate Memory, *RBANS DM* Repeatable Battery for the Assessment of Neuropsychological Status: Delayed Memory, *(W)* Word, *(C)* Colour, *(CW)* Colour-Word, *SRL-SRS* Self-Regulation of Learning Self-Report Scale, *SRQ* Self-Regulation Questionnaire

The results of MANOVA comparing the results of the three groups showed a significant difference between the open-skilled, closed-skilled, and no physical activity participation groups for cognitive function assessment scores [*F* (34,82) = 2.14, *P* = .003; partial η^2^ = .46) (Table 4). Statistically significant mean scores were observed for the Benton Judgement of Line Orientation (*P* = .001), Stroop (C) (*P* < .05), and Stroop (CW) (*P* < .05). Significant better results (*P* < .05) were detected in the open-skilled physical activity group when compared with the no physical activity group in the Benton Judgement of Line Orientation, Stroop (CW), Stroop (W), SRL-SRS (Evaluation) and borderline significance (*P* = .070) was found for Trail Making B. Significant better results were detected in the closed-skilled when compared with the no physical activity groups for the Benton Judgement of Line Orientation (*P* < .001), Stroop (C) (*P* < .05) and Digit Span Forward (*P* < .05). There was borderline significance (*P* = .079) in Stroop (CW) between the open- and closed-skilled groups. No significant results were found for any of the other assessments.

**Table 4:** MANOVA Results of Between-Subjects Effects for Open-Skilled, Closed-Skilled and No Physical Activity Groups

Dependent Variable	Without Adjustments				Adjusted for Age				Adjusted for Gender				Adjusted for Age and Gender			
	*P = .003*	η^2^ = .46	Significant Pairwise Comparisons (*p-value)*		*P = .003*	η^2^ = .47	Significant Pairwise Comparisons (*p-value)*		*P = .002*	η^2^ = .48	Significant Pairwise Comparisons –*(p-value)*		*P = .002*	η^2^ = .48	Significant Pairwise Comparisons *(p-value)*	
RBANS IM Index	.575	.019			.726	.023			.170	.084			.276	.086		
RBANS DM Index	.813	.007			.675	.026			.195	.078			.231	.094		
Trail Making A	.505	.023			.323	.059			.516	.039			.373	.072		
Trail Making B	.125	.069	Open	No PA = .070#	.031*	.143			**.027**	.148	Open	No PA = .049*	.008*	.214	Open	No PA = .067#
Benton Judgement of Line Orientation	.001**	.230	Closed	No PA < .001**	.001**	.262	Closed	No PA < .001**	<.001**	.270	Closed	No PA < .001**	<.001**	.298	Closed	No PA < .001**
			Open	No PA = .006*			Open	No PA = .004*			Open	No PA = .006*			Open	No PA = .004*
Digit Span Forward	.126	.069	Closed	No PA = .045	.229	.072	Closed	No PA = .052#	.174	.083	Closed	No PA = .038*	.269	.087	Closed	No PA = .044*
Digit Span Backward	.427	.029			.639	.029			.639	.029			.795	.029		
Stroop (W)	.083	.082	Open	No PA = .032*	.120	.097	Open	No PA = .027*	.130	.094	Open	No PA = .030*	.167	.134	Open	No PA = .024*
Stroop (C)	.039*	.106	Closed	No PA = .015*	.024*	.151	Closed	No PA = .021*	.039*	.136	Open	No PA = .078#	.158 .026*	.110 .177	Closed	No PA = .027*
											Closed	No PA = .020*				
Stroop (CW)	.019*	.128	Open	No PA = .006*	.033*	.141	Open	No PA = .007*	.038*	.136	Open	No PA = .005*	.058# .058#	.148 .148	Open	No PA = .007*
			Open	Closed = .079#			Open	Closed = .078#			Open	Closed = .068#				
SRL-SRS																
– Planning	.646	.015			.833	.015			.789	.018			.903	.018		
– Self-Monitoring	.300	.041			.444	.046			.468	.043			.595	.048		
– Evaluation	.124	.069	Open	No PA = .043*	.185	.081	Open	No PA = .052#	.127	.094	Open	No PA = .037*			Open	No PA = .045*
– Reflection	.358	.035			.467	.043			.528	.038			.602	.047		
– Effort	.347	.036			.364	.054			.550	.036			.528	.054		
– Self-Efficacy	.243	.048			.410	.049			.394	.051			.554	.052		
SRQ	.366	.034			.549	.036			.332	.058			.483	.059		

*Open* Open-skilled physical activity group, *Closed* Closed-skilled physical activity group, *No PA* No physical activity group, *RBANS IM* Repeatable Battery for the Assessment of Neuropsychological Status: Immediate Memory, *RBANS DM* Repeatable Battery for the Assessment of Neuropsychological Status: Delayed Memory, *W* Word, *C* Colour, *CW* Colour-Word, *SRL-SRS* Self-Regulation of Learning Self-Report Scale, *SRQ* Self-Regulation Questionnaire

*P* = Probability based on α=0.05, significant values in bold; **Indicates significance at the 0.001 level and *Indicates significance at the 0.05 level. #Indicates borderline significance

^ Participation in Physical Activity includes number of years of engagement in physical activity, total number of hours and total number of sessions of engagement per week

The results of MANCOVA showed a significant overall effect when adjusted for age [F (34,82) = 2.11, *P* = .003; partial η^2^ = .47]; adjusted for sex [F (34,82) = 2.21, *P* = .002; partial η^2^ = .48] and adjusted for both age and sex [F (34,80) = 2.18, *P* = .002; partial η^2^ = .48] (Table 4). Adjusting for the possible differences in either age or sex, significant findings for Trail Making B, Benton Judgement of Line Orientation Scores, Stroop (C) and Stroop (CW) were found among the three groups. When adjusted for age, significant differences were found between open-skilled and no physical activity groups in Benton Judgement of Line Orientation (*P* = .004) and Stroop (C) (*P* = .007), and between closed-skilled and no physical activity groups in Benton Judgement of Line Orientation (*P* < .001) and Stroop (C) (*P* = .021). When adjusted for sex, significant differences were found between open-skilled and no physical activity groups in Trail Making B (*P* = .001), Benton Judgement of Line Orientation (*P* = .006) and Stroop (CW) (*P* = .005), and between closed-skilled and no physical activity groups in Benton Judgement of Line Orientation (*P* < .001), Digit Span Forward (*P* = .038) and Stroop (C) (*P* = .020). However, when both age and sex controls were used together, only borderline significant (*P* = .058) was found in Stroop (CW) between the open-skilled and no physical activity groups. Similar between-group differences (*P* < .05) were observed in all other cognitive functions when adjusted for age and sex as compared with the results when adjusted for age only. The between-group differences among open-skilled and no physical activity groups for Stroop (C) (*P* = .078) and between-group differences regarding closed-skilled and open-skilled groups for Stroop (CW) (*P* = .068) were only borderline significant when controlled for sex, and not significant when age and sex controls were used together.

When comparing the cognitive function of participants in the open- and closed-skilled physical activity group, the results of MANCOVA showed a non-significant overall effect when adjusted for age, sex and participation in physical activity [F (17,11) = .857, *P* = .624; partial η^2^ = .57] (Table 5). Significant differences were found between open- and closed-skilled physical activity groups in Trail Making B (*P* = .044), Stroop (W) (*P* = .019) and Stroop (C) (*P* = .045). Participants in the open-skilled physical activity group had better results in Trail Making B and Stroop (W), while those in the closed-skilled physical activity group had a better result in Stroop (C).

**Table 5:** MANCOVA Results of Between-Subjects Effects for Open-Skilled and Closed-Skilled Physical Activity Groups

Dependent Variable	Adjusted for Age, Sex and ^@^Participation in Physical Activity	
	*P = .624*	η^2^ = .57
RBANS IM Index	.373	.200
RBANS DM Index	.149	.279
Trail Making A	.758	.111
Trail Making B	.044*	.361
Benton Judgement of Line Orientation	.473	.175
Digit Span Forward	.790	.103
Digit Span Backward	.306	.219
Stroop (W)	.019*	.408
Stroop (C)	.045*	.360
Stroop (CW)	.181	.264
SRL-SRS		
Planning	.960	.050
Self-Monitoring	.103	.306
Evaluation	.398	.194
Reflection	.930	.063
Effort	.283	.227
Self-Efficacy	.657	.134
SRQ	.052#	.351

*P* = Probability based on α=0.05, significant values in bold; *Indicates significance at the 0.05 level. #Indicates borderline significance

^@^ Participation in Physical Activity includes number of years of engagement in physical activity, total number of hours and total number of sessions of engagement per week

*RBANS IM* Repeatable Battery for the Assessment of Neuropsychological Status: Immediate Memory, *RBANS DM* Repeatable Battery for the Assessment of Neuropsychological Status: Delayed Memory, *(W)* Word, *(C)* Colour, *(CW)* Colour-Word, *SRL-SRS* Self-Regulation of Learning Self-Report Scale, *SRQ* Self-Regulation Questionnaire

## Discussion

The purpose of this cross-sectional study was to understand how engagement in open- and closed-skilled forms of physical activity affect the cognitive function of older adults. Findings from this study suggest that physical activity, in both open- and closed-skilled forms, and a higher level of participation intensity and frequency, may be beneficial for specific cognitive function in older adults.

While not all results were significant, in accordance with our first hypothesis, overall mean scores across each of the cognitive function assessments showed a better performance for either the open- or closed-skilled participants compared with the no-physical activity group. Significant between-group differences for visuospatial perception, processing speed, selective attention, inhibition and self-regulation of evaluation were found between either the open-skilled and no physical activity groups, or closed-skilled and no physical activity groups, adding further to the large body of evidence showing better cognitive function ability for older adults who participate in physical activity compared with those who do not [3, 4, 8, 11, 17, 30, 62, 63].

Results showed that, in comparison to closed-skilled and non-physical activity, open-skilled physical activity was associated with significantly better selective attention and inhibition skills (Stroop (CW)). When the sample was controlled for age, sex and both age and sex, as well as the participation in physical activity, the open-skilled group continued to demonstrate significantly better attention and inhibition skills as well as showing better visual scanning and cognitive flexibility (Trail Making Test B) when compared to the other participant groups. Previous research has found positive links between physical activity participation, irrespective of the form, and better Stroop [8] and Trail Making Test B scores in older adults [4]; the finding of better inhibition ability in the open-skilled group mirrors the results of studies of younger populations [37–39, 42] and those of Huang et al.’s [17] study of older adults, which indicated that engagement in open-skilled exercise types was associated with greater neural efficiency for tasks that demanded interference control. Thus, the strength of the open-skilled physical activity group’s scores for Stroop (CW) and in the Trail Making Test B may be explained by the theory that more cognitively demanding tasks, such as open-skilled physical activity, may result in better inhibition and cognitive flexibility in older adults [10, 12, 17, 64]. However, further investigation of this theory is necessary to confirm these benefits.

Additionally, in this study, closed-skilled physical activity was significantly associated with better selective attention ability (Stroop (C)) and better visuospatial perception (Benton Judgement of Line Orientation). These findings are consistent with previous research that has demonstrated positive associations between the overall level of physical activity engagement by older adults and both attention skills [7, 8, 65] and visuospatial perception [66]. Nevertheless, it is not clear why the closed-skilled group outperformed the open-skilled group in these specific areas as previous research has found the opposite [31, 67]. As a result, our second hypothesis was not supported. It is possible that these results are due to the particular types of closed-skilled physical activity in the sample as research has suggested that there may be selectively beneficial effects of physical activity on cognitive function depending on the inherent qualities of the particular activity type [17]. In our sample, in addition to swimming, which demands attention to specific positions of the arms, legs, head, and torso for greater swimming performance [32], one of the most common forms of closed-skilled physical activity was Tai Chi. This martial art is understood to be beneficial for cognitive function [68, 69] including attention [70] and visuospatial processing owing to the task demands of learning choreographed sequences of movements [68].

In partial support of our third hypothesis, increased number of hours of physical activity in the participant sample was positively associated with visuospatial perception, working memory, selective attention, and inhibition ability, while an increased number of years of physical activity engagement and physical activity sessions per week was positively associated with visuospatial perception and selective attention ability. In addition to further supporting the benefits of greater frequency and intensity of physical activity for cognitive functions in older adults [3, 71], these findings may also indicate that participants who engaged in more frequent physical activity had increased fitness levels, which has in itself been shown to benefit cognitive function [72, 73]. Furthermore, previous research has also found there to be a dose-response relationship between moderate-to-vigorous-intensity physical activity and better cognitive function ability in older adults [35, 71]. The present study’s results indicate that hours of physical activity engagement had a particularly strong impact on cognitive function scores compared with the other measures of physical activity intensity and frequency in this study. However, further investigation as to why this was the case, and reasons for why not more of the assessment results, including all self-regulation measures, were affected by physical activity levels of intensity and frequency remains necessary.

While this study addressed a considerable range of cognitive function indicators, it did not find significant correlations between physical activity type or frequency of participation across a number of key cognitive functions. Notably, no significant effects were found for immediate and delayed memory. Although some studies report benefits to memory from physical activity [2, 7, 8, 11], others have similarly inconclusive results [63, 74]. For example, while 16 studies included in a review by Smith et al. [63] determined there to be modest effects to memory from aerobic exercise, the twelve studies looking specifically at the impact on working memory ability found no benefits. Nevertheless, there was one significant finding in the present study showing correlations between hours of physical activity participation and working memory. Chang et al. [75] also found better working memory to be associated with higher physical activity levels. However, unlike our study, which categorised individual physical activity patterns, physical activity levels were measured as a combined score of intensity, duration, and frequency. Therefore, it does not help to explain why working memory in our study was only significantly correlated with the number of hours of physical activity per week but not sessions per week or years or participation. Further research may be able to determine whether physical activity intensity has a stronger impact on working memory function in older adults when compared to the frequency of physical activity.

The only significant finding for self-regulation ability in this study was identified in the SRL-SRS (Evaluation). Analysis of between-group differences showed better results for the open-skilled group when compared with the no-physical activity group. There is limited evidence in previous literature to explain this finding. Nevertheless, it may be the case that the open-skilled group employed more successful metacognitive strategies by using evaluative skills in their behaviour. This might be explained by the specific task demands of open-skilled physical activity. For example, golf, one of the most commonly engaged in forms of open-skilled physical activity in the study sample, requires participants to consistently evaluate the changing environment of the game and consequently make numerous conscious adjustments to their movements [76]. However, it is not clear why no other self-regulation ability was significant for either the open-skilled or closed-skilled group. Therefore, our study has not determined there to be overall significant effects on self-regulation ability in older adults as a result of physical activity. It is possible that administering two self-regulation surveys towards the end of the assessment session, and in addition to the five other cognitive assessments, may have placed too much cognitive demand on participants and caused participant burden [48]. As a result, self-regulation measures may have been affected. Future investigation should consider strategies for administering multiple assessments while avoiding the likelihood of participant fatigue to prevent possible influence on assessments.

There were several limitations to acknowledge in our study. Firstly, to fulfil the inclusion criteria, participants self-reported information regarding their cognitive and physical health. As a result, this study can only assume that the information provided was accurate. As demonstrated in other studies [17, 30, 44], further investigation of this research area should require participants to complete a standardised cognitive assessment, such as the Mini-Mental State Examination (MMSE) [77], prior to data collection to screen participants’ cognition. Because our study did not do this, cognitive function measures might have been negatively impacted as it was not possible to exclude participants who may have had mild cognitive impairments or other neurological conditions. Comprehensive screening of participants’ physical health and fitness levels would also allow for a more detailed analysis of the true impact of physical activity types and intensity. Implementing these screening methods would allow for improved certainty of cognitive and fitness level homogeneity in the sample, thus increasing the validity of findings. This study did not investigate the effects of other personal factors such as schooling and type of job. Cognitive function may be affected by these factors other than the engagement of physical activity.

Moreover, as this study is a cross-sectional analysis, conclusions of causality between open-skilled and closed-skilled physical activity and cognitive function cannot be adequately determined from these results. The purpose of this study has been to provide additional data to the currently limited body of knowledge of this research area. However, further research, which establishes greater cause and effect probability by embarking on good quality intervention studies, using larger sample sizes and controlling for the personal factors and the level of physical activity participation [2, 78], is required. This type of research design is necessary in order to properly understand the benefits of different physical activity types on cognitive function ability in older adults.

## Conclusions

In summary, this preliminary study suggests that, in accordance with previous research, physical activity may have selectively beneficial effects on cognitive function [17, 41]. Similar to the findings of Dai et al. [30], Huang et al. [17] and Guo et al. [44], results show that physical activity, regardless of being open- or closed-skilled, provides benefits for older adults’ cognitive function ability when compared with no physical activity participation, with some potential additional advantages provided as a result of open-skilled physical activity engagement for selected cognitive processes. In our study, open-skilled physical activity was associated with better inhibition, visual tracking, and cognitive flexibility while closed-skilled physical activity was associated with better selective attention and visuospatial perception. However, the majority of self-regulation ability was not significantly influenced by open- or closed-skilled physical activity in this study. Importantly, physical activity intensity and frequency were significantly correlated with a range of cognitive functions. Although much further investigation is required for a thorough understanding of this area, this study contributes important findings of the benefits of both open- and closed-skilled physical activity participation by older adults for a range of cognitive function measures.

## Data Availability

The datasets used and/or analyzed during the current study are available from the corresponding author on reasonable request.

## Abbreviations

RBANS: Repeatable Battery for the Assessment of Neuropsychological Status
SRL-SRS: Self-Regulation of Learning Self-Report Scale
SRQ: Self-Regulation Questionnaire
Stroop (C): Stroop (Colour)
Stroop (CW): Stroop (Colour-Word)
Stroop (W): Stroop (Word)
